# Differences in modifiable factors of oral squamous cell carcinoma in the upper and lower of oral fissure

**DOI:** 10.18632/oncotarget.20520

**Published:** 2017-08-24

**Authors:** Lingjun Yan, Fa Chen, Fengqiong Liu, Yu Qiu, Jing Wang, Junfeng Wu, Xiaodan Bao, Zhijian Hu, Xiane Peng, Xu Lin, Lin Cai, Lisong Lin, Baochang He

**Affiliations:** ^1^ Department of Epidemiology and Health Statistics, Fujian Provincial Key Laboratory of Environment Factors and Cancer, School of Public Health, Fujian Medical University, Fujian, China; ^2^ Key Laboratory of Ministry of Education for Gastrointestinal Cancer, Fujian Medical University, Fujian, China; ^3^ Fujian Key Laboratory of Tumor Microbiology, Fujian Medical University, Fujian, China; ^4^ Department of Oral and Maxillofacial Surgery, The First Affiliated Hospital of Fujian Medical University, Fujian, China; ^5^ Laboratory of Facial Plastic and Reconstruction of Fujian Medical University, Fujian, China; ^6^ Laboratory Center, The Major Subject of Environment and Health of Fujian Key Universities, School of Public Health, Fujian Medical University, Fujian, China

**Keywords:** oral squamous cell carcinoma, oral fissure, modifiable factors, alcohol consumption, case-control study

## Abstract

The aim of this study was to explore differences in the effects of modifiable factors on oral squamous cell carcinoma (OSCC) occurring in the lower oral fissure (LOF) and upper oral fissure (UOF). We conducted a case-control study with 697 OSCC patients (119 UOF and 578 LOF) and 1910 frequency-matched controls in Fujian province, China. Data on demographic characteristics and possible modifiable factors was collected using a structured questionnaire. Unconditional logistic regression was utilized to calculate the odds ratios (ORs) and corresponding 95% confidence intervals (CIs). Alcohol drinking was more strongly associated with an increased risk of OSCC-LOF than OSCC-UOF. Tobacco smoking, the number of teeth lost ≥5, wearing denture, and recurrent oral ulceration showed similarly associations with OSCC-LOF and -UOF risk. Similarly, the beneficial effects of tea consumption, tooth-brushing ≥2times per day, high intake of fresh fish, seafood, green-leafy vegetables, other vegetables and fruits were not significantly different on OSCC-LOF and -UOF. Although most of the modifiable factors exert similar effects on both OSCC sites, this study suggests that the sites of oral cavity in LOF may be affected more by alcohol drinking than the sites in UOF. Further studies with larger samples are warranted to confirm our findings.

## INTRODUCTION

Oral squamous cell carcinoma (OSCC), the most common malignancy of the oral cavity, is a growing health problem in the world [1]. As reported, various anatomical sites of oral cavity showed different incidence rates of OSCC. Tongue is considered as the most frequently affected site, followed by gingiva, buccal mucosa, floor of mouth, palate and lip, and occasionally found in retromolar area or other oral sites [2]. Furthermore, survival varies in relation to tumor sites in origin [3]. Thus, it is essential to assess site-specific risk factors for OSCC.

The cause of OSCC is multifactorial. In terms of extrinsic factors, tobacco smoking and alcohol drinking have been established two major risk factors for OSCC [4]. Moreover, a previous study found that the relative risk associated with smoking for OSCC was the highest in the retromolar area followed by the floor of mouth and buccal mucosa, while alcohol drinkers had significantly higher risk in floor of mouth than tongue [5]. Diet is the most frequently exposed factor in daily life since oral cavity is access of the digestive tract. High consumption of fruits, vegetables and fresh fish have been shown to exert protective effects on the overall oral cavity of cancer [6, 7]. However, epidemiological evidences on other food items and the risk of OSCC are inconsistent. Additionally, Velly *et al* [8] demonstrated that oral sores secondary to ill-fitting dentures was primarily associated with an increased risk of tongue neoplasms, and poor oral hygiene, eg little tooth brushing was considered to related to high risks of cancers in tongue and other parts of oral cavity.

It is conceivable that these modifiable factors may have different effects on different anatomical sites of oral cavity. According to a recent report [9], the proportions of OSCC occurring in the lower oral fissure (LOF) are higher than that of the upper oral fissure (UOF), which is consistent with our clinical observations. However, up to date, little information is available to demonstrate the differences in modifiable factors between OSCC-LOF and OSCC-UOF.

Therefore, we performed a hospital-based case-control study in southeast of China to assess modifiable factors and the risk of two groups of OSCC-LOF and -UOF, respectively. And then, we further compared the differences of such factors between the two groups which may provide an explanation for the different incidence in OSCC-LOF and -UOF.

## RESULTS

Table 1 shows the demographic characteristics of two case groups of both OSCC-LOF and -UOF and controls. The distributions of age, gender, education level and residence were similar between case groups and controls (P>0.05). There were significant differences among the groups with regard to the distributions of family history of cancer and BMI (P<0.05).

**Table 1:** Baseline characteristics of case and control subjects

Characteristics	Controls (%) (n=1910)	OSCC-UOF^a^ (%) (n=119)	OSCC-LOF ^b^ (%) (n=578)	*χ*^*2*^	*P*-value
Age (years)				2.437	0.296
≤60	1039 (54.40)	58 (48.74)	326 (56.40)		
>60	871 (45.60)	61 (51.26)	252 (43.60)		
Gender				0.857	0.651
Male	1287 (67.38)	81 (68.07)	378 (65.40)		
Female	623 (32.62)	38 (31.93)	200 (34.60)		
Education level				1.844	0.765
Illiterate	201 (10.52)	14 (11.76)	61 (10.55)		
Primary and middle school	1258 (65.87)	81 (68.07)	370 (64.01)		
High school and above	451 (23.61)	24 (20.17)	147 (25.43)		
Residence				4.647	0.098
Rural	1104 (57.80)	75 (63.03)	311 (53.81)		
Urban	806 (42.20)	44 (36.97)	267 (46.19)		
BMI (kg/m^2^)^a^				111.444	<0.001
18.5-23.9	1053 (55.13)	79 (66.39)	367 (63.49)		
<18.5	99 (5.18)	15 (12.60)	85 (14.71)		
≥24	758 (39.69)	25 (21.01)	126 (21.80)		
Family history of cancer				18.344	<0.001
No	1693 (88.64)	102 (85.71)	473 (81.83)		
Yes	217 (11.36)	17 (14.29)	105 (18.17)		

^a^oral squamous cell carcinoma occurs on the upper oral fissure

^b^oral squamous cell carcinoma occurs on the lower oral fissure

As shown in Table 2, tobacco smoking significantly increased the risk of OSCC: the adjusted ORs were 3.02(95%CI: 1.73-5.26) for OSCC-UOF and 2.27(95%CI: 1.73-2.99) for OSCC-LOF. Moreover, a strong dose-response relationship was found between tobacco smoking intensity and OSCC-UOF or OSCC-LOF risk (all P_trend_ <0.001). Alcohol drinking was only significantly associated with the risk of OSCC-LOF (OR= 2.42, 95% CI 1.91-3.08), but not in OSCC-UOF (OR= 1.53, 95%CI: 0.97-2.41). Furthermore, our case-case studies showed that alcohol drinking was found more strongly associated with an increased risk of OSCC-LOF than OSCC-UOF (OR=1.74, 95%CI: 1.04-2.92). Additionally, tea consumption showed a decreased risk of either OSCC group.

**Table 2:** Adjusted odds ratios and 95%CIs for OSCC by tobacco smoking, alcohol drinking and tea consumption

Variables		Case–control comparisons				Case–case comparisons
	Controls	OSCC-UOF		OSCC-LOF		OSCC-LOF VS OSCC-UOF
	n	n	*OR*(95%*CI*)^a^	n	*OR*(95%*CI*)^a^	*OR*(95%CI)^a^
Tobacco smoking^b^						
No	1211	53	1.00	279	1.00	1.00
Yes	699	66	3.02 (1.73-5.26)	299	2.27 (1.73-2.99)	0.66 (0.35-1.26)
Tobacco smoking intensity (pack-years)^b^						
Never	1211	53	1.00	280	1.00	1.00
<20	196	6	1.05 (0.41-2.72)	50	1.43 (0.96-2.11)	1.19 (0.43-3.30)
20-40	241	27	3.79 (2.00-7.21)	94	2.06 (1.46-2.90)	0.48 (0.23-1.00)
≥40	262	33	3.97 (2.12-7.44)	154	3.09 (2.25-4.23)	0.67 (0.33-1.38)
P-trend			<0.001		<0.001	0.418
Alcohol drinking^c^						
No	1449	77	1.00	338	1.00	1.00
Yes	461	42	1.53 (0.97-2.41)	240	2.42 (1.91-3.08)	1.74 (1.04-2.92)
Quantity of alcohol drinking (g/d)^c^						
Never	1449	77	1.00	338	1.00	1.00
<20	217	11	0.93 (0.47-1.84)	65	1.52 (1.09-2.11)	1.72 (0.82-3.61)
20-60	139	14	1.63 (0.85-3.10)	79	2.56 (1.83-3.58)	1.79 (0.89-3.59)
≥60	105	17	2.66 (1.43-4.95)	96	4.19 (2.98-5.90)	1.73 (0.89-3.36)
P-trend			0.006		<0.001	0.063
Tea consumption^d^						
No	1075	75	1.00	356	1.00	1.00
Yes	835	44	0.61 (0.40-0.93)	222	0.61 (0.49-0.76)	1.03 (0.66-1.61)
Duration of tea consumption (years)^d^						
No	1076	75	1.00	356	1.00	1.00
<25	364	11	0.54 (0.31-0.94)	60	0.49 (0.37-0.65)	0.93 (0.52-1.69)
≥25	470	33	0.68 (0.41-1.12)	162	0.74 (0.57-0.96)	1.10 (0.65-1.86)
P-trend			0.09		0.002	0.930
Quantity of tea consumed (ml/day)^d^						
No	1076	75	1.00	356	1.00	1.00
<700	605	33	0.63 (0.40-0.99)	166	0.64 (0.51-0.81)	1.03 (0.64-1.66)
≥700	229	11	0.56 (0.28-1.11)	56	0.54 (0.38-0.76)	1.04 (0.50-2.16)
P-trend			0.09		<0.001	0.930

^a^Adjustment for age, gender, education level, residence, BMI, family history of cancer

^b^Additionally adjusted for alcohol drinking

^c^Additionally adjusted for tobacco smoking

^d^Additionally adjusted for tobacco smoking, alcohol drinking

Table 3 reveals the effects of oral hygiene related variables on OSCC. In case-control comparisons, an inversed association was observed between tooth-brushing ≥2 times/day and the risk of OSCC-UOF or OSCC-LOF, the ORs were 0.62(95%CI: 0.41-0.94) and 0.67(95%CI: 0.54-0.83), respectively. In contrast, number of missing teeth ≥5, wearing denture and recurrent dental ulceration showed significant increased risks in two groups, and seems to display stronger associations with OSCC-LOF risk although no statistically significant differences between OSCC-UOF and -LOF. Additionally, regular dental visits showed protective potential effect in relation to the risks of OSCC-LOF only.

**Table 3:** Adjusted odds ratios and 95%CIs for OSCC by oral hygiene

Variables	Case–control comparisons					Case–case comparisons
	Controls	OSCC-UOF		OSCC-LOF		OSCC-LOF VS OSCC-UOF
	n	n	*OR* (95%*CI*)^a^	n	*OR* (95%*CI*)^a^	*OR* (95%CI)^a^
Tooth-brushing (times/day)						
<2	1025	80	1.00	365	1.00	1.00
≥2	885	39	0.62(0.41-0.94)	213	0.67(0.54-0.83)	1.11(0.71-1.73)
Teeth lost						
None	711	30	1.00	152	1.00	1.00
<5	501	31	1.46(0.86-2.49)	168	1.74(1.34-2.26)	1.06(0.61-1.86)
≥5	698	58	1.88(1.12-3.16)	258	2.12(1.62-2.77)	0.96(0.56-1.64)
P-trend			0.010		<0.001	0.752
Wearing denture						
No	1172	59	1.00	293	1.00	1.00
Yes	738	60	1.50(1.01-2.24)	285	1.61(1.31-1.98)	1.03(0.67-1.57)
Regular dental visits (years/time)						
None	1526	102	1.00	503	1.00	1.00
>5	204	10	0.77(0.38-1.52)	37	0.56(0.38-0.82)	0.75(0.36-1.58)
≤5	180	7	0.58(0.26-1.27)	38	0.49(0.34-0.72)	0.97(0.42-2.28)
P-trend			0.146		<0.001	0.891
Recurrent dental ulcer						
No	1865	108	1.00	513	1.00	1.00
Yes	45	11	4.58(2.27-9.26)	65	5.79(3.83-8.75)	1.16(0.58-2.32)

^a^Adjustment for age, gender, education level, residence, BMI, family history of cancer, tobacco smoking, alcohol drinking.

The associations between dietary factors and OSCC risk are summarized in Table 4. Subjects who consumed domestic meat ≥3 times per week had approximate 40% lower odds of OSCC cancer (OR= 0.58, 95%CI: 0.34-0.98 for OSCC-UOF; OR= 0.62, 95%CI: 0.48-0.80 for OSCC-LOF),compared to those who consumed <3 times per week. Additionally, high intake of fresh fish, seafood, green-leafy vegetables, other vegetables and fruits were significantly associated with reduced risks of OSCC, and the associations were similar between OSCC-UOF and OSCC-LOF. Consuming milk and dairy products ≥1time/week and eggs ≥5 times/week decreased the risk of OSCC-LOF, but were not significantly associated with OSCC-UOF risk. Other food items such as red meat, beans and soy products and pickled food were not significantly related to either OSCC-UOF or-LOF. Regarding the results of case-case comparisons, the effects of these dietary factors were not significantly different between OSCC-UOF and -LOF.

**Table 4:** Adjusted odds ratios (ORs) and 95%CIs for OSCC by dietary factors

Variables	Case–control comparisons					Case–case comparisons
	Controls	OSCC-UOF		OSCC-LOF		OSCC-LOF VS OSCC-UOF
	n	n	*OR* (95%*CI*)^a^	n	*OR* (95%*CI*)^a^	*OR* (95%CI)^a^
Red meat (per week)						
<5times	909	58	1.00	286	1.00	1.00
≥5times	1001	61	0.97(0.66-1.43)	292	0.83(0.68-1.01)	0.92(0.61-1.39)
Domestic meat (per week)						
<3times	1473	102	1.00	492	1.00	1.00
≥3times	437	17	0.58(0.34-0.98)	86	0.62(0.48-0.80)	1.03(0.58-1.82)
Fish (per week)						
<3times	772	67	1.00	303	1.00	1.00
≥3times	1138	52	0.56(0.38-0.81)	275	0.60(0.49-0.73)	1.09(0.73-1.63)
Seafood (per week)						
<3times	1222	94	1.00	443	1.00	1.00
≥3times	688	25	0.49(0.31-0.78)	135	0.52(0.41-0.65)	1.05(0.64-1.72)
Green-leafy vegetables (per day)						
<1time	78	22	1.00	98	1.00	1.00
≥1time	1832	97	0.20(0.12-0.33)	480	0.22(0.16-0.31)	1.02(0.60-1.71)
Other vegetables (per day)						
<1time	112	25	1.00	122	1.00	1.00
≥1time	1798	94	0.25(0.15-0.41)	456	0.26(0.19-0.35)	0.93(0.57-1.53)
Fruits (per week)						
<3times	746	82	1.00	383	1.00	1.00
≥3times	1164	37	0.31(0.20-0.47)	195	0.33(0.27-0.41)	1.07(0.68-1.68)
Milk and dairy products (per week)						
<1time	1042	75	1.00	383	1.00	1.00
≥1time	868	44	0.74(0.50-1.11)	195	0.59(0.48-0.73)	0.80(0.52-1.24)
Eggs (per week)						
<5times	1218	88	1.00	401	1.00	1.00
≥5times	692	31	0.65(0.42-1.00)	177	0.74(0.60-0.92)	1.16(0.73-1.84)
Beans and soy products (per week)						
<1time	584	28	1.00	178	1.00	1.00
≥1time	1326	91	1.42(0.91-2.21)	400	0.91(0.73-1.12)	0.63(0.40-1.01)
Pickled food (per week)						
<1time	1362	79	1.00	396	1.00	1.00
≥1time	548	40	1.25(0.84-1.86)	182	1.08(0.87-1.33)	0.93(0.61-1.43)

^a^Adjustment for age, gender, education level, residence, BMI, family history of cancer, tobacco smoking, alcohol drinking.

## DISCUSSION

To our knowledge, the current study is the first to differentiate the effects of modifiable factors on OSCC-UOF and -LOF. One important novel finding was that alcohol drinking was more closely associated with OSCC-LOF risk than OSCC-UOF. Additionally, we also observed that tobacco smoking, the number of teeth lost≥5, wearing denture, and recurrent dental ulceration were risk factors for both OSCC-UOF and -LOF. Likewise, the protect effects of tea consumption, tooth-brushing ≥2times per day, high intake of fresh fish, seafood, vegetables, and fruits showed no differences between OSCC-UOF and -LOF.

Interestingly, the present study demonstrated that alcohol consumers were more prone to develop OSCC-LOF, which is in line with a previous study [5], showing the highest ORs to be seen in the floor of mouth followed by the retromolar area for alcohol drinkers. A possible mechanism for this could attribute to the different physiological characteristics of anatomical sites of oral cavity. Alcohol has been speculated to increase the risk of OSCC by dissolving the lipid component of the epithelium and increasing the permeability of the mucosa. While, compared with the hard palate and gingiva, the permeability of the buccal mucosa, the lateral border of tongue and the floor of the mouth is much greater, resulting in the oral mucosa of LOF to be more vulnerable to other carcinogenic toxins [10]. Additionally, consistent with many epidemiological studies, our study also confirmed tobacco smoking significantly increased the risk of both OSCC sites [11, 12]. Carcinogenic components in tobacco smoke, such as N-nitroso compounds, polycyclic aromatic hydrocarbons (PAH) and carbon monoxide could make the tumor cells of OSCC more aggressive [13] and induce specific mutations [14].

Recurrent oral ulceration is a common oral mucosal inflammatory lesion. A recent study reported that recurrent oral ulceration have increased the risk of oral cancer in Chinese women by nearly 5-fold [15]. The present study further observed that the OR of recurrent oral ulceration in the LOF was a bit greater than that of UOF although the difference was not statistically significant. Rivera et al. [16] also found that the frequency of oral mucosal lesions in tongue appears to be similar to that of the palate (18.4% VS 16.6%). Abdullah [17] and Safadi [18] indicated the most common site of recurrent oral ulceration was lips and buccal mucosae, followed by tongue. Recurrent oral ulceration was considered as an early precancerous lesions of oral cancer [19]. However, there is no literature to clarify the mechanism of recurrent oral ulcers for OSCC-UOF and -LOF which still needs further study.

The anticancer effects of tea consumption on different anatomical sites of oral cancer were reported *in vivo* [20], *in vitro* [21] and epidemiological studies [22, 23]. Our data also showed tea consumption could protect against both OSCC-UOF and -LOF. This might be explained by the fact that epigallocatechin gallate (EGCG), the main anti-carcinogenic components of tea inhibits the invasion and growth of oral carcinoma cell [24]. Regarding for dietary factors, high intake of vegetables and fruits affected the risk of OSCC similarly in the UOF and LOF. It was also observed in previous study that the beneficial effects of vegetables and fruits seemed to be no significantly different between tongue and other sites of mouth [6]. Vegetables and fruits are rich in vitamin C, carotenoids, folic acid and other antioxidants, all of these constituents are reported to be of anti-cancer effects [25].

There are several limitations in this study. First, as a retrospective case-control study, recall bias should be concerned. In order to minimize recall bias, all patients were newly diagnosed, and the definitions of lifestyle variables were clearly given during the interview. Second, this study only compared the major modifiable lifestyle risk factors between OSCC-UOF and -LOF. Therefore, other factors such as HPV infection, betel chewing, oral flora and genetic factors should be also considered in future studies. Third, we only collected the frequency of each food item which may not reflect standard amounts per day/week. Hopefully, the effects of dietary factors were estimated according to the quantity of food intake in the future studies. Fourth, the sample size of the OSCC-UOF group is relatively small, which may limit the statistical power of this study. Further studies with larger samples are warranted to confirm our findings.

In conclusion, although most of the modifiable factors show similar important for the etiology of OSCC-UOF and -LOF, the present study suggests that alcohol drinking may increase the risk of OSCC-LOF more strongly than the risk of OSCC-UOF. This finding may provide an additional understanding of the higher incidence of OSCC in LOF.

## MATERIALS AND METHODS

### Study participants

From September 2010 to December 2016, a hospital-based case-control study was carried out in Fujian province, China. As described previously [26], eligible cases were histologically confirmed primary OSCC patients and consecutively recruited from The First Affiliated Hospital of Fujian Medical University. Second primary cancer, recurrent and metastasized tumor were excluded. According to lesion site, cases were stratified into two groups: (1) OSCC occurs on the upper oral fissure (OSCC-UOF), including palate, upper lip, upper gum and upper buccal; (2) OSCC occurs on the lower oral fissure (OSCC-LOF), including tongue and tongue base, floor of mouth, lower lip, lower gum, lower buccal and retromolar area. There was no overlap between the two groups. Of the 759 individuals diagnosed with OSCC, 62 patients were excluded because of uncertain classification. Therefore, 697 eligible cases (including 119 UOF and 578 LOF) were enrolled in this study.

During the same period as cases, 1910 controls were randomly selected from medical examination center in the same hospital. They were free of any malignant disease. Controls and cases were frequency matched by age and gender. The recruiting rates were 98.1% for cases and 91.7% for controls. All participants signed an informed consent after told about the purpose of this research. The present study was performed in accordance with the ethical standards laid down in the 1964 Declaration of Helsinki and its later amendments and was approved by the Institutional Review Board of Fujian Medical University (Fuzhou, China).

### Data collection

Face to face interviews were conducted by well-trained interviewers to obtain participants’ information using a structured questionnaire. Information collected included demographic characteristics, family history of cancer, main lifestyle such as tobacco smoking, alcohol drinking and tea consumption and dietary habits, and oral hygiene related variables.

Subjects were considered tobacco smokers if they had smoked at least 100 cigarettes during their lifetime. Alcohol drinkers were defined as those had consumed at least one drink per week for more than 6 months continuously. Those who had consumed at least 1 cup of tea per week continuously for at least 6 months were considered tea drinkers. Detailed information on oral hygiene was recorded as follows: tooth brushing/day, the number of missing teeth, wearing of dentures (no/yes), regular dental visits (years/time) and recurrent dental ulcer (no/yes).

Regarding to dietary intake, participants were asked about the intake frequency of 10 broad categories food (red meat; domestic meat; fish; seafood; milk and dairy products; eggs; green-leafy vegetables and other vegetables; fruits; beans and soy products; pickled food). The intake frequency options of each food item were: 3 times per day; 2 times per day; 1 time per day; 5-6 times per week, 3-4 times per week; or 1-2 times per week; less than 1 time per week or not at all. The questionnaire referred to food intake during the previous 12 months.

### Statistical analysis

The distributions of demographic characteristics between two case groups and controls were compared by chi square test. The continuity variables and each dietary item were grouped according to the median of the controls. Unconditional logistic regression models were used to estimate odds ratios (ORs) and corresponding 95% confidence intervals (CIs) for the relationship between modifiable lifestyle factors and OSCC risk in both case-control and case-case comparisons. Wald chi square test was used to test for trends by treating categorical variables as continuous factors in the regression model. All analyses were conducted using R software version 3.1.1. Statistical significance was considered at *P* < 0.05 (two-sided).

## References

[1] Chi AC, Day TA, Neville BW (2015). Oral cavity and oropharyngeal squamous cell carcinoma--an update. CA Cancer J Clin.

[2] Chen F, Cao Y, Huang J, Yan L, Lin L, Liu F, Liu F, Wu J, Qiu Y, Cai L, He B (2017). A novel prognostic index for oral squamous cell carcinoma patients with surgically treated. Oncotarget.

[3] Liu F, Chen F, Huang J, Yan L, Liu F, Wu J, Qiu Y, Zheng X, Zhang R, Lin L, He B (2017). Prospective study on factors affecting the prognosis of oral cancer in a Chinese population. Oncotarget.

[4] Radoi L, Paget-Bailly S, Cyr D, Papadopoulos A, Guida F, Schmaus A, Cenee S, Menvielle G, Carton M, Lapotre-Ledoux B, Delafosse P, Stucker I, Luce D (2013). Tobacco smoking, alcohol drinking and risk of oral cavity cancer by subsite: results of a French population-based case-control study, the ICARE study. Eur J Cancer Prev.

[5] Jovanovic A, Schulten EA, Kostense PJ, Snow GB, van der Waal I (1993). Tobacco and alcohol related to the anatomical site of oral squamous cell carcinoma. J Oral Pathol Med.

[6] Franceschi S, Barra S, La Vecchia C, Bidoli E, Negri E, Talamini R (1992). Risk factors for cancer of the tongue and the mouth. A case-control study from northern Italy. Cancer.

[7] Chen F, Lin T, Yan L, Liu F, Huang J, Liu F, Wu J, Qiu Y, Lin L, Cai L, He B (2017). Novel polymorphism in FADS1 gene and fish consumption on risk of oral cancer: a case-control study in southeast China. Oncotarget.

[8] Velly AM, Franco EL, Schlecht N, Pintos J, Kowalski LP, Oliveira BV, Curado MP (1998). Relationship between dental factors and risk of upper aerodigestive tract cancer. Oral Oncol.

[9] Zini A, Czerninski R, Sgan-Cohen HD (2010). Oral cancer over four decades: epidemiology, trends, histology, and survival by anatomical sites. J Oral Pathol Med.

[10] Squier CA, Cox P, Wertz PW (1991). Lipid content and water permeability of skin and oral mucosa. J Invest Dermatol.

[11] Wang X, Xu J, Wang L, Liu C, Wang H (2015). The role of cigarette smoking and alcohol consumption in the differentiation of oral squamous cell carcinoma for the males in China. J Cancer Res Ther.

[12] Maasland DH, van den Brandt PA, Kremer B, Goldbohm RA, Schouten LJ (2014). Alcohol consumption, cigarette smoking and the risk of subtypes of head-neck cancer: results from the Netherlands Cohort Study. BMC Cancer.

[13] Bundgaard T, Bentzen SM, Sogaard H (1995). Histological differentiation of oral squamous cell cancer in relation to tobacco smoking. Eur J Cancer B Oral Oncol.

[14] Sudbo J (2004). Novel management of oral cancer: a paradigm of predictive oncology. Clin Med Res.

[15] Chen F, He BC, Yan LJ, Qiu Y, Lin LS, Cai L (2017). Influence of oral hygiene and its interaction with standard of education on the risk of oral cancer in women who neither smoked nor drank alcohol: a hospital-based, case-control study. Br J Oral Maxillofac Surg.

[16] Rivera C, Droguett D, Arenas-Marquez MJ (2017). Oral mucosal lesions in a Chilean elderly population: A retrospective study with a systematic review from thirteen countries. J Clin Exp Dent.

[17] Abdullah MJ (2013). Prevalence of recurrent aphthous ulceration experience in patients attending Piramird dental speciality in Sulaimani City. J Clin Exp Dent.

[18] Safadi RA (2009). Prevalence of recurrent aphthous ulceration in Jordanian dental patients. BMC Oral Health.

[19] Scully C, Kirby J (2014). Statement on mouth cancer diagnosis and prevention. Br Dent J.

[20] Zhao X, Qian Y, Zhou YL, Wang R, Wang Q, Li GJ (2014). Pu-erh tea has *in vitro* anticancer activity in TCA8113 cells and preventive effects on buccal mucosa cancer in U14 cells injected mice *in vivo*. Nutr Cancer.

[21] Hua Y, Jianhua L, Qiuliang W, Jun F, Zhi C (2006). Effects of tea polyphenols on telomerase activity of a tongue cancer cell line: a preliminary study. Int J Oral Maxillofac Surg.

[22] Chen F, He BC, Yan LJ, Liu FP, Huang JF, Hu ZJ, Lin Z, Zheng XY, Lin LS, Zhang ZF, Cai L (2017). Tea consumption and its interactions with tobacco smoking and alcohol drinking on oral cancer in southeast China. Eur J Clin Nutr.

[23] Chen F, Yan L, Lin L, Liu F, Qiu Y, Liu F, Huang J, Wu J, Cai L, Cai G, Aoyagi K, He B (2017). Independent and joint effects of tea and milk consumption on oral cancer among non-smokers and non-drinkers: a case-control study in China. Oncotarget.

[24] Hsu SD, Singh BB, Lewis JB, Borke JL, Dickinson DP, Drake L, Caughman GB, Schuster GS (2002). Chemoprevention of oral cancer by green tea. Gen Dent.

[25] Harasym J, Oledzki R (2014). Effect of fruit and vegetable antioxidants on total antioxidant capacity of blood plasma. Nutrition.

[26] Chen F, Yan L, Liu F, Huang J, Liu F, Wu J, Qiu Y, Zheng X, Cai L, Lin L, He B (2016). Oral human papillomavirus infection, sexual behaviors and risk of oral squamous cell carcinoma in southeast of China: a case-control study. J Clin Virol.

